# Impact of Patient Age on Clinical Efficacy and Toxicity of Checkpoint Inhibitor Therapy

**DOI:** 10.3389/fimmu.2021.786046

**Published:** 2021-11-16

**Authors:** Selina K. Wong, Caroline A. Nebhan, Douglas B. Johnson

**Affiliations:** Department of Medicine, Vanderbilt University Medical Center and Vanderbilt Ingram Cancer Center, Nashville, TN, United States

**Keywords:** age, geriatric, PD-1, Nivolumab, Pembrolizumab, ipilimumab, toxicity, young

## Abstract

The addition of immune checkpoint inhibitors (ICIs) to the therapeutic armamentarium for solid malignancies has resulted in unprecedented improvements in patient outcomes in many cancers. The landscape of ICIs continues to evolve with novel approaches such as dual immune checkpoint blockade and combination therapies with other anticancer agents including cytotoxic chemotherapies and/or antiangiogenics. However, there is significant heterogeneity seen in antitumor responses, with certain patients deriving durable benefit, others experiencing initial benefit followed by acquired resistance necessitating change in therapy, and still others who are primarily refractory to ICIs. While generally better tolerated than traditional cytotoxic chemotherapy, ICIs are associated with unique toxicities, termed immune-related adverse events (irAEs), which can be severe or even lethal. As a disease of aging, older individuals make up a large proportion of patients diagnosed with cancer, yet this population is often underrepresented in clinical trials. Because ICIs indirectly target malignant cells through T cell activation, it has been hypothesized that age-related changes to the immune system may impact the efficacy and toxicity of these drugs. In this review, we discuss differences in the clinical efficacy and toxicity of ICIs in patients at the extremes of age.

## Introduction

Since the development and successful application of immune checkpoint inhibitors (ICIs) across various cancer types, efforts to understand predictors of response to ICIs and likelihood of developing toxicities have been ongoing. Patients treated with ICIs can exhibit vastly different responses, with complete, durable responses on one end of the spectrum and primary resistance to therapy on the other end. The development of toxicities is similarly variable between patients, with no reliable predictors of severe immune-related adverse events (irAEs).

Cancer is classically understood as a “disease of aging,” as rates of cancer within a population generally increase with age (1). Despite this, data on how patients at the extremes of age respond to ICIs are scarce. Older patients are more likely to be excluded from participation in clinical trials, largely due to exclusionary comorbidities, prior cancer diagnoses, and reduced functional status (2, 3). Within the United States, patients over the age of 65 account for nearly 60% of cancer incidence, but only approximately 40% of cancer clinical trial participation. This incongruence is amplified with increasing age; patients over the age of 80 account for 16% of cancer incidence, but only 4% of cancer clinical trial participants (4). In the context of chemotherapy, studies have suggested that older patients more frequently have co-morbidities and are often treated less aggressively, but have similar outcomes to younger patients when fit enough to undergo comparable treatment (5). When compared to traditional cytotoxic chemotherapy, ICIs generally have a more favorable toxicity profile, making these therapies a potentially appealing option for older patients with limited functional reserve. Here, we aim to discuss age-related changes to the immune system that may impact ICI efficacy and toxicity, efficacy of ICIs in younger *versus* older patients, and age-specific considerations of ICI toxicities.

## Aging and the Immune System

Aging is the most significant non-modifiable risk factor for cancer development. It has been postulated to impact ICI efficacy and toxicity. The mechanism of action for ICIs largely relies on the patient’s own immune system to reach a balance between ability to mount an effective anti-cancer response and risk of autoimmunity resulting in potentially severe irAEs. Immunosenescence, conceptually defined as the declining function of the immune system with increasing age, could lead to a differential response to ICIs between age groups. This topic has been most extensively explored in the context of adaptive immune response to vaccines in older adult patients; numerous studies have demonstrated decreased or insufficient antibody responses after common vaccines given to patients over the age of 60 (6–8). However, research in this area has been limited by lack of consensus definition as well as validated methods to measure immunosenescence. Importantly, an individual’s chronological age is not necessarily reflective of a patient’s physiological age or underlying immune system. The point at which the immune system demonstrates changes associated with being “old” is not only variable between individuals, but also between populations as a result of underlying genetics, lifestyle, and pathogenic exposures (9).

Immunosenescence is characterized by a decrease in peripheral naïve T cells with a relative increase in memory T cells (10, 11), decrease in T cell receptor repertoire (12–14), and changes in the composition of regulatory T cell populations (15). Memory T cells generated from aged naïve T cells demonstrate lower proliferation rates and effector cytokine production, thus resulting in an inferior immune response compared to those generated from young naïve T cells (16). Epigenetic alterations in aging immune cells and cancer cells also play a role in anti-cancer response, with studies of epigenetic biomarkers and combined epigenetic therapy with immunotherapy underway (17–19). Targeted pathway inhibition, however, could potentially reverse these changes in preclinical studies (20). Taken together, immunosenescence is thought to result in a decreased ability to respond to antigenic stimulation and simultaneous, paradoxical chronic low-level inflammation and autoimmunity (21).

## Efficacy

Despite the striking proliferation of ICI-based clinical trials in the past decade, there remains a gap in knowledge regarding the impact of age on the efficacy of ICIs. The ability to draw conclusions from clinical trial data is limited by the small proportion of older participants, all of whom are also highly selected for fitness and unlikely representative of the general older population. Existing knowledge on ICIs in older patients has been derived from data across tumor types and drawn from pooled meta-analyses of clinical trials, retrospective studies, and case reports. We have summarized key outcomes from select phase III clinical trials in **Tables 1**–**3**. Although ultimately inconclusive on the basis of subgroup analyses of data from single clinical trials, we have highlighted trends suggestive of age-related differences in ICI efficacy in the tables.

**Table 1:** Select phase III trials of ICIs in advanced disease.

Cancer type	Study	Treatment	Number of patients	Key outcome(s), HR (95%CI)		
PD-1/PD-L1 Inhibitors						
NSCLC	Keynote 024 Reck et al. (22)	Pembrolizumab *vs* chemotherapy	Total – 305	PFS		OS
			<65 - 141	0.61 (0.40-0.92)		0.60 (0.38-0.96)
			≥65 - 164	0.45 (0.29-0.70)^#^		0.64 (0.42-0.98)
Melanoma	Checkmate 066 Robert et al. (23)	Nivolumab *vs* chemotherapy	Total – 418	OS		
			<65 - 200	0.52 (0.32-0.85)		
			≥65 and <75 - 151	0.44 (0.24-0.81)		
			≥75 - 67	0.25 (0.10-0.61)^#^		
Colorectal (MSI-H/dMMR)	KEYNOTE-177 André et al. (24)	Pembrolizumab *vs* chemotherapy	Total - 307	PFS		
			≤70 – 217	0.52 (0.37-0.75)		
			>70 - 90	0.77 (0.46-1.27)^^^		
PD-1 + CTLA-4 Inhibitors						
Renal cell	Checkmate 214 Motzer et al. (25)	Nivolumab + ipilimumab *vs* sunitinib	Total – 847	OS (among intermediate and high-risk pts)		
			<65 - 524	0.53 (0.40-0.71)		
			≥65 and <75 - 258	0.86 (0.58-1.27)^^^		
			≥75 - 65	0.97 (0.48-1.95)^^^		
NSCLC	Checkmate 227 Hellmann et al. (26)	Nivolumab + ipilimumab *vs* chemotherapy	Total – 1166	OS		
			<65 - 611	0.70 (0.58-0.85)		
			≥65 and <75 - 442	0.76 (0.61-0.95)		
			≥75 - 113	0.84 (0.55-1.29)^^^		
Melanoma	Checkmate 067 Larkin et al. (27)	Nivolumab + ipilimumab *vs* nivolumab	Total – 630	5-year PFS rate	5-year OS rate	
			<65 - 383	0.73 (0.56-0.94)	0.80 (0.60-1.06)	
			≥65 - 247	0.89 (0.65-1.23)^^^	0.86 (0.62-1.20)	
Melanoma	Checkmate 067 Larkin et al. (27)	Nivolumab + ipilimumab *vs* ipilimumab	Total – 629	5-year PFS rate	5-year OS rate	
			<65 - 367	0.41 (0.31-0.52)	0.48 (0.37-0.63)	
			≥65 - 262	0.44 (0.33-0.59)	0.59 (0.43-0.81)^^^	

^#^Stronger effect/lower HR in older patients.

^^^Weaker effect/higher HR in older patients.

NSCLC, non-small cell lung cancer; MSI-H, microsatellite instability-high; dMMR, deficient mismatch repair; PFS, progression-free survival; OS, overall survival.

**Table 2:** Select phase III trials of chemoimmunotherapy and ICI-targeted therapy in advanced disease.

Cancer type	Study	Treatment	Number of patients	Key outcome(s), HR (95% CI)	
PD-1/PD-L1 Inhibitor + Chemotherapy					
NSCLC	Keynote 407 Paz-Ares et al. (28)	Pembrolizumab + chemotherapy *vs* chemotherapy	Total – 559	OS	
			<65 - 254	0.52 (0.34-0.80)	
			≥65 - 305	0.74 (0.51-1.07)^^^	
NSCLC	Keynote 189 Gandhi et al. (29)	Pembrolizumab + chemotherapy *vs* chemotherapy	Total – 616	OS	
			<65 - 312	0.43 (0.31-0.61)	
			≥65 - 304	0.64 (0.43-0.95)^^^	
SCLC	IMpower133 Horn et al. (30)	Atezolizumab + chemotherapy *vs* chemotherapy	Total – 403	OS	
			<65 - 217	0.92 (0.64-1.32)	
			≥65 - 186	0.53 (0.36-0.77)^#^	
Breast (TNBC)	IMpassion130 Schmid et al. (31)	Atezolizumab + chemotherapy *vs* chemotherapy	Total – 902	PFS	
			18-40 - 114	0.79 (0.53-1.16)	
			41-64 - 569	0.84 (0.70-1.01)	
			≥65 - 219	0.69 (0.51-0.94)^#^	
Bladder	IMvigor130 Galsky et al. (32)	Atezolizumab + chemotherapy *vs* chemotherapy	Total – 851	PFS	
			<65 - 306	0.82 (0.63-1.06)	
			≥65 - 545	0.80 (0.66-0.97)^#^	
PD-1/PD-L1 Inhibitor + Targeted Therapy					
Melanoma	IMspire150 Gutzmer et al. (33)	Atezolizumab + vemurafenib + cobimetinib *vs* vemurafenib + cobimetinib	Total – 514	PFS	
			<65 - 394	0.82 (0.64-1.06)	
			≥65 - 120	0.63 (0.40-0.99)^#^	
Renal	KEYNOTE-426 Rini et al. (34)	Pembrolizumab + axitinib *vs* sunitinib	Total – 861	PFS	OS
			<65 - 538	0.70 (0.54-0.90)	0.47 (0.30-0.73)
			≥65 - 323	0.63 (0.45-0.81)	0.59 (0.36-0.97)^^^
Renal	Checkmate 9ER Choueiri et al. (35)	Nivolumab + cabozantinib *vs* sunitinib	Total – 651	PFS	OS
			<65 - 401	0.44 (0.33-0.58)	0.44 (0.29-0.67)
			≥65 - 250	0.68 (0.48-0.98)^^^	0.90 (0.56-1.44)^^^
Renal	JAVELIN Renal 101 Motzer et al. (36)	Avelumab + axitinib *vs* sunitinib	Total – 560	PFS	
			<65 - 354	0.60 (0.44-0.81)	
			≥65 - 206	0.71 (0.46-1.09)^^^	

^#^Stronger effect/lower HR in older patients.

^^^Weaker effect/higher HR in older patients.

NSCLC, non-small cell lung cancer; SCLC, small-cell lung cancer; TNBC, triple negative breast cancer; PFS, progression-free survival; OS, overall survival.

**Table 3:** Select phase III trials of ICIs as adjuvant or neoadjuvant therapy.

Cancer type	Study	Treatment	Number of patients	Key outcome(s), HR (95% CI)	
NSCLC	PACIFIC Antonia et al. (37)	Durvalumab *vs* placebo	Total – 713	PFS	OS
(Adjuvant)			<65 - 391	0.43 (0.32-0.57)	0.62 (0.44-0.86)
			≥65 - 322	0.74 (0.54-1.01)^^^	0.76 (0.55-1.06)^^^
Melanoma	EORTC 18071 Eggermont et al. (38)	Ipilimumab *vs* placebo	Total – 951	RFS	OS
(Adjuvant)			<50 - 425	0.68 (0.49-0.94)	0.64 (0.43-0.96)
			51 - 64 - 358	0.84 (0.60-1.19)^^^	0.78 (0.51-1.20)^^^
			≥65 - 168	0.80 (0.49-1.30)^^^	0.88 (0.50-1.56)^^^
Renal	KEYNOTE-564 Choueiri et al. (39)	Pembrolizumab *vs* placebo	Total – 994	DFS	
(Adjuvant)			<65 - 664	0.62 (0.45-0.84)	
			≥65 - 330	0.84 (0.56-1.26)^^^	
Breast (TNBC)	KEYNOTE-522 Schmid et al. (40)	Pembrolizumab + chemotherapy *vs* chemotherapy	Total – 602	Difference in pCR	
(Neoadjuvant)			<65 - 531	12.2 (3.4-21.0)	
			≥65 - 71	22.3 (-2.1-43.5)^^^	

^#^Stronger effect/lower HR in older patients.

^^^Weaker effect/higher HR in older patients.

NSCLC, non-small cell lung cancer; TNBC, triple negative breast cancer; PFS, progression-free survival; OS, overall survival; RFS, relapse-free survival; DFS, disease-free survival; Pcr, pathological complete response.

To elucidate the impact of age on efficacy of ICIs, several meta-analyses have been performed with 65 and 75 years as the most commonly used age cut-offs to differentiate between older versus younger patients. One of the largest meta-analyses was reported by Huang et al. in 2019, including 34 studies and containing over 20,000 patients across various advanced tumor types. Survival analyses were done using subgroup analyses with cutoffs of either 65 years and 75 years, depending on each individual study. ICIs were associated with statistically significant improvement in overall survival (OS) in patients <65 years, ≥ 65 years, and <75 years compared to their respective control groups, but less so for patients ≥75 years (HR 0.88 compared with control for ≥75 years, 95%CI 0.67-1.16, p=0.377). As for progression-free survival (PFS), improvement over control groups was seen for patients <65 years and ≥ 65 years, but not in subgroup analyses for patients <75 years or ≥75 years. Importantly, the authors noted that analyses of the ≥75 year patients were limited by the relatively small number of patients in this age cohort (~3%), and relatively fewer studies using age of 75 years as a cutoff for subgroup analyses (41). Several other meta-analyses have also demonstrated no statistically significant difference in treatment efficacy of ICI-based therapy between younger and older patients using the age cut-off of 65 years (7, 8, 42, 43), with potentially less benefit among patients 75 years and older (6, 44–47).

Real-world retrospective analyses have similarly demonstrated comparable outcomes across age groups with respect to efficacy and safety, although drawing definitive conclusions remains challenging given the different age cut-offs used across studies and the relatively small proportion of patients over the age of 70-75 (48–50). A large retrospective review of 410 patients treated with single-agent ICI across lung cancer, melanoma, and genitourinary cancer (150 patients aged 70 or older and 185 patients 69 or younger) found no significant difference between age groups in regard to PFS, OS, or grade 3 or higher irAEs (49). Ibrahim et al. evaluated older patients with a retrospective single-institution cohort study of 99 patients aged 75 and up (median age of 80 years) treated with ICI monotherapy for metastatic melanoma, demonstrating effectiveness and safety despite advanced age (51). More recently, a study of 45 patients 80 years or older (median age of 85) with non-small cell lung cancer (NSCLC) similarly determined single-agent ICI to be a reasonable option with disease control rate of 60% and PFS of 3.4 months (52). Also, among very elderly patients, a retrospective study by our group found that NSCLC, melanoma and genitourinary cancer patients aged ≥85 years experienced similar efficacy of single-agent checkpoint inhibitors when compared to patients aged 80-85 years. Objective response rate among NSCLC (n=276), melanoma (n=280), and genitourinary cancer patients (n=126) over the age of 80 years was 32%, 39% and 26%, respectively (53). Data among nonagenarian patients become even more limited. One case report summarizes the clinical course of three patients over the age of 90 treated for metastatic melanoma: two patients treated with single-agent ICI and one patient who received combination ICI. Although one patient required high-dose corticosteroids for grade 2 hepatitis while on combination nivolumab and ipilimumab, she was able to resume single-agent nivolumab following resolution of the irAE. Among the three patients, two achieved complete or partial response while one other had prolonged stable disease (54).

The specific ICI target axis (PD-1/PD-L1 *versus* CTLA-4) may potentiate the effect of age on treatment efficacy due to underlying changes associated with immunosenescence. A pooled analysis of 24 randomized trials by Ninomiya et al. found no age-dependent difference in survival benefit from ICIs in patients younger *versus* older than 65 years (HR 0.76 *versus* 0.78, p=0.82), but subgroup analyses evaluating the impact of ICI type suggested less survival benefit for older patients compared to their younger counterparts among those treated with an anti-CTLA-4 ICI (HR 0.90 *vs* 0.77, p=0.26). This difference in efficacy between the two age groups was not seen with PD-1/PD-L1 inhibitors, HR 0.74 *vs* 0.74, p=0.96 (42). While the CTLA-4 pathway is thought to operate earlier in the immune response by regulating autoimmunity at the initial stage of naïve T cell activation, PD-1 regulates already activated T cells in peripheral tissues (55). Among older patients, thymic involution, decreased naïve T cell output, and ultimately lower levels of circulating naïve T cells may contribute to a differential age-related survival benefit between CTLA-4 and PD-1/PD-L1 inhibitors (11, 56).

Finally, there have been relatively sparse data regarding extremely young patients. Small prospective and retrospective studies have suggested that pediatric patients may respond to ICIs, including with CNS tumors, lymphoma, and solid tumors (57–59). One study of pembrolizumab showed that 9 of 15 pediatric patients with Hodgkin lymphoma responded to treatment, broadly similar to adult data. Responses across a range of PD-L1-positive solid tumors though, were infreuqent in this study (59). It is unclear whether age is related to these findings, or whether other factors like histology or low mutational burden play a larger role. Similarly, there have been few studies in younger adults (e.g., under 40 years), so it remains unclear whether ICI has comparable efficacy in this population. The generally high response rates generated by ICI in many cancers that affect young adults (melanoma, MSI-high cancers, Hodgkin lymphoma) suggest that many patients do experience responses.

## Toxicity

Despite the success of ICIs, the risks of irAEs remain an important consideration in the assessment and counselling of patients for therapy (60). The risk, type, and severity of toxicities are variable depending on individual patient characteristics as well as the ICI regimen used. Up to 10-30% of patients can develop a grade 3 or higher irAE with single-agent PD-1/PD-L1 or CTLA-4 inhibitors, and this increases to over 50% for those receiving combination ICI therapy (61). Though mild to moderate irAEs can often be managed with the initiation of systemic corticosteroids and/or discontinuation of the offending ICI, severe irAEs can require additional immunosuppressive therapies and are potentially life-threatening.

A 2021 update of the FDA adverse event reporting system found an increased rate of irAEs in adults ages ≥65 as compared to 18-64 (41.55% *versus* 33.3%) among those treated with ICI monotherapy or combinations (62). Data from a retrospective analysis suggested that patients more likely to suffer fatal irAEs tended to be older (median age of 70 *vs* 62 years) (63). A retrospective case-control study of patients with melanoma, renal cell carcinoma or NSCLC compared 185 patients <65 years, 154 patients 65-74 years and 109 patients ≥75 years. This study found no significant difference in any-grade irAE rates between age cohorts. Endocrine toxicity was found to be more common in patients <65 years, while dermatologic toxicity was more common in patients ≥75 years. Interestingly, older patients were found to be less likely to discontinue ICI treatment due to toxicity (discontinuation rate 7.4% among patients ≥75 years *versus* 20.5% among patients <65 years, p=0.006) (64). Additional retrospective series have suggested that rheumatologic irAEs are more common in older patients, whereas hepatitis and colitis may be more prevalent in younger patients (48). Shah et al. found a similar rates of any-grade irAE, but decreased rate of severe toxicity and hospitalization rate in older adults with melanoma as compared to younger patients. Older adults hospitalized for irAEs, however, experienced longer hospital stays and increased risk of death from irAE. This finding was confirmed with a validation pharmacoviligance dataset (65).

Prompted by concerns that chronologic age alone does not adequately predict treatment tolerance, geriatric-specific assessment indices have been developed to better stratify older adult patients into distinct functional groups for the purpose of predicting treatment tolerance. Developed by geriatricians, these indices assess functional status, comorbidities, cognitive function, nutritional status, psychological state, social support, and concurrent medications, and may be patient- or provider-administered (66, 67). Previously developed tools have demonstrated validity in stratifying older adult patients into predicted outcome groups based upon treatment with traditional cytotoxic chemotherapy and/or radiation, but such tools have not yet been well validated in the era of immunotherapy (68). One small study of 28 older cancer patients has suggested that a high prevalence of impairment in geriatric assessment domains is associated with shorter duration of treatment with ICI, although it was unclear whether this correlated with increased irAEs or worse survival outcome (69). Sarcopenia, which is more common in older adults, has also been reported as correlating with inferior outcomes in many cancer therapies. However, studies are conflicting whether this correlates with ICI responses (70–72).

Another consideration for geriatric adults is whether they have sufficient functional reserve to recover from severe toxicities. Several common irAEs may impose substantial physiologic strain and critical illness in some cases (e.g., pneumonitis, colitis, myocarditis), potentially limiting some patients’ ability to recover. High-dose steroids, the treatment for severe irAEs, also carry the risks of delirium, arrhythmias, hyperglycemia, and infection, all of which may particularly impact older patients.

When weighing the potential risks and benefits of ICIs, there are unique challenges faced by younger patients that are distinct from those of their older counterparts. ICI-related infertility is one such issue that has recently garnered attention, albeit still an area that is not well studied. Fertility can be affected by primary hypogonadism (via direct impacts on the gonads such as orchitis and impaired spermatogenesis/oogenesis) or secondary hypogonadism as a result of hypophysitis (73). ICI-mediated inflammation of the gonads can uncommonly result in hypogonadism and possibly impaired spermatogenesis, as several cases of epididymo-orchitis (unilateral and bilateral) have been reported (74–76). Subclinical injury may also occur in the absence of overt inflammation; a retrospective cohort autopsy study by Scovell et al. of 13 men (median age of 54, range 23-78 years) with metastatic melanoma found that 6 out of 7 men who received ICI therapy had histopathologic evidence of impaired spermatogenesis compared to 2 of 6 men who were treatment-naïve (77).

Infertility can also result from dysregulation of the pituitary gland. Hypophysitis is an irAE that appears to be more common with anti-CTLA-4 agents than PD-1/PD-L1 inhibitors (78), with rates of hypophysitis up to 11% among individuals receiving ipilimumab (79, 80). Given the critical role of the pituitary in downstream hormone regulation and endocrine homeostasis, its impairment carries the risk of premature menopause in women and impaired sperm production in men.

Pregnancy is another challenging situation unique to younger patients with cancer treated with ICIs. Of note, PD-L1 is highly expressed on syncytiotrophoblast cells in the placenta and likely plays a critical role in maintaining fetal tolerance (81). There is only limited evidence for outcomes apart from several case reports of pregnancy during ICI therapy (82–84). While there have been case reports of inadvertent but successful conception during treatment with ICI, the United Stated Food and Drug Administration (FDA) has assigned pregnancy category C (risk not ruled out) to ipilimumab, category D (positive evidence of risk) to pembrolizumab, and category X (contraindicated in pregnancy) for durvalumab (other FDA-approved ICIs remain unassigned at time of publication). Because melanoma is the most common malignancy diagnosed during pregnancy, further studies dedicated to understanding this patient population are needed (85).

## Conclusion

ICIs appear to have comparable efficacy for younger and older patients, although meta-analysis and retrospective data suggest that the magnitude of benefit may be smaller in those over the age of 75 and in the setting of anti-CTLA-4 ICIs, potentially due to underlying changes associated with immunosenescence. Younger patients have particular considerations related to toxicities, including infertility and contraception. Given the expanding role of ICIs in the armamentarium of cancer therapies, targeted clinical trials are needed to prospectively deepen our understanding of these agents in older adults.

## Author Contributions

All authors contributed to writing and editing the manuscript.

## Funding

Funded by the Susan and Luke Simons Directorship in Melanoma Fund.

## Conflict of Interest

DJ advises/consults for BMS, Catalyst, Jansen, Iovance, Merck, Mosaic BioEngineering, Novartis, Oncosec, Pfizer, and Targovax, and receives research funding from BMS and Incyte.

The remaining authors declare that the research was conducted in the absence of any commercial or financial relationships that could be construed as a potential conflict of interest.

## Publisher’s Note

All claims expressed in this article are solely those of the authors and do not necessarily represent those of their affiliated organizations, or those of the publisher, the editors and the reviewers. Any product that may be evaluated in this article, or claim that may be made by its manufacturer, is not guaranteed or endorsed by the publisher.
